# Shrimp Alpha-2-Macroglobulin Prevents the Bacterial Escape by Inhibiting Fibrinolysis of Blood Clots

**DOI:** 10.1371/journal.pone.0047384

**Published:** 2012-10-17

**Authors:** Vorrapon Chaikeeratisak, Kunlaya Somboonwiwat, Anchalee Tassanakajon

**Affiliations:** Department of Biochemistry, Faculty of Science, Center of Excellence for Molecular Biology and Genomics of Shrimp, Chulalongkorn University, Bangkok, Thailand; University Medical Center Utrecht, The Netherlands

## Abstract

Proteomic analysis of the hemocytic proteins of *Penaeus monodon* (*Pm*) has previously shown that alpha-2-macroglobulin (A2M) was among the proteins that showed substantially altered expression levels upon *Vibrio harveyi* infection. Therefore, in this study its potentially important role in the response of shrimp to bacterial infection was further characterized. The yeast two-hybrid system revealed that the receptor binding domain of *Pm*A2M interacted with the carboxyl-terminus of one or both of the transglutaminase type II isoforms, which are key enzymes involved in the shrimp clotting system. In accord with this, *Pm*A2M was found to be localized on the extracellular blood clots and to colocalize with clottable proteins. RNA interference (RNAi)-mediated knockdown of A2M transcript levels reduced the *Pm*A2M transcript levels (∼94%) and significantly reduced the bacterial seizing ability of the clotting system, resulting in an up to 3.3-fold higher number of *V. harveyi* that systemically disseminated into the circulatory system at 5 min post-infection before subsequent clearance by the immune system. Furthermore, an appearance of *Pm*A2M depleted clots in the presence of *V. harveyi* strikingly demonstrated fibrinolysis zones surrounding the bacteria. This study provides the first evidence of the vital role of *Pm*A2M in enhancing bacterial sequestration by protecting blood clots against fibrinolysis.

## Introduction

Infection of *Vibrio harveyi*, one of the major species categorized in the genus *Vibrio* [1], in penaeid shrimp causes necrosis of the basal tissues followed by the systemic dissemination of the bacteria [2]. The characteristic aspects of *V. harveyi* diseased penaeid shrimp are a glowing in the dark (luminous vibriosis) and the occurrence of developmental disorders of the epidermal tissues obstructing the digestive tract (bolitas negrican). Although the pathogenic mechanisms of *V. harveyi* and its associated phages have not been clearly elucidated, extracellular products, such as proteases, phospholipase and hemolysin, are regarded as the major virulent factors produced by the bacteria [3]. Indeed, proteases secreted from diverse pathogenic organisms have been recognized as a significant factor that plays a variety of crucial roles in facilitating the invasion and dissemination of the pathogen into the host, and in evasion of the immune system and alteration of housekeeping functions [4]. In addition to many cysteine proteases, considered as the major toxins, three alkaline metal ion sensitive proteases have also been characterized from *V. harveyi*, and these exotoxins exert lethal pathogenesis to penaeid shrimp, and in particular to the black tiger shrimp, *Penaeus monodon* [5]–[10].

Consequentially, as expected, specific protease inhibitors are a vital element of the host to defend and neutralize against invasion by protease-secreting pathogens so as to diminish the illness and death among the hosts. From these, a number of protease inhibitors that are ubiquitously found in microorganisms and multicellular organisms have been found and they serve essential roles in controlling both the endogenous and exogenous proteases [11]. These protease inhibitors can be divided into two main classes; (i) active site inhibitors, which directly interact with the active site of the endopeptidase, and (ii) the alpha-2-macroglobulins (A2M), which inhibit the protease activity via a conformation change to engage the targeted protease inside the A2M molecule conformation and so result in an irreversible inhibition called the physical entrapment mechanism [12], [13].

In invertebrates, A2M is a broad range proteinase inhibitor and a highly abundant plasma protein [14] that is crucially involved in many immune responses, such as phagocytosis in the hard tick, *Ixodes ricinus* [15], and in the mosquito, *Anopheles gambiae* [16], the prophenoloxidase activating system in the crayfish, *Pacifastacus leniusculus* [17], and the blood clotting system in *P. leniusculus* [18] and the horseshoe crab, *Limulus polyphemus* [19]. In penaeid shrimp, the cloning, biochemical characterization, binding studies and expression at both the transcription and translation levels of A2M have been reported [20]–[28]. The protein expression profile in the major immune tissues of *V. harveyi* infected *P. monodon* revealed that *Pm*A2M was the most strongly altered protein in hemocytes [28] and lymphoid organ [29], following *V. harveyi* infection. However, to date there is no direct evidence concerning the biological role(s) of A2M in response to severe pathogens, and especially in response of shrimp to *V. harveyi*.

This research, therefore, focused on which immune pathways A2M is involved in and how A2M functions against *V. harveyi* infections in *P. monodon*. The yeast two-hybrid (Y2H) technology was firstly conducted to identify interacting partners of the receptor binding domain (RBD) of *Pm*A2M. Since the RBD is exposed upon protease inactivation, it should be noted that this study reveals only one part of the roles of *Pm*A2M in the immune response against *V. harveyi*. The immunofluorescent technique was later performed in order to localize *Pm*A2M in different hemocyte types and the hemolymph clots of shrimp. Furthermore, based on the fact that the pathogen entrapment within hemolymph clots acts as an early immune mechanism in other invertebrates [30], the biological roles of *Pm*A2M in sequestration of *V. harveyi* within the shrimp blood clots were also investigated using RNA interference (RNAi).

## Materials and Methods

### Experimental animals and *V. harveyi* 639 cultivation

Healthy black tiger shrimp, *P. monodon*, each weighing approximately either 15 grams or 1–3 g body weight were used in the analysis of *Pm*A2M protein expression levels as well as the localization of *Pm*A2M protein and in RNAi based gene silencing experiments, respectively. Shrimp were acclimatized in artificially aerated sea water (15 ppt salinity) for a week before experimental use. The shrimp pathogenic *V. harveyi* strain 639 was freshly prepared, as previously reported [31].

### Ethics statement

The experimental shrimp were obtained from the Broodstock and Larval Development Research Center at Walailuk University, Nakornsrithammarat Province, Thailand, where is not privately-owned or protected. No specific permits were required for the described field studies and for these locations. We confirm that these field studies did not involve endangered or protected species.

The manual of Standard Operating Procedures (SOPs) for the Ethical Use of Live Aquatic Animals approved by the Museum Victoria Animal Ethics Committee on 12^th^ November 2010 was applied to our studies, since there have been no official recommendations for the experimental use of invertebrates for scientific purposes in Thailand. The transporting, raising and experimental processing of the animals were strictly performed as described in the manual of SOPs. Also, the humane killing at the end of experiments was carried out by first completely anaesthetizing the experimental shrimp and then placing them into a −20°C freezer, and all efforts were taken in order to minimize animal suffering, as pointed out in the SOPs.

### Screening for the *Pm*A2M interacting protein(s) from shrimp hemocytes using a Y2H assay

The RBD of *Pm*A2M was amplified from the hemocyte cDNA of unchallenged shrimp using the gene specific forward and reverse primers (Table 1), with 5′ flanking *Nde*I and *Bam*HI sequences (underlined), respectively, and the Advantage®2 Polymerase Mix (Clontech). The purified amplicon was then cloned into the bait vector, pGBKT7 (Clontech). The resulting recombinant plasmid (pGBKT7-RBD-A2M) was introduced into the yeast *Saccharomyces cerevisiae* strain Gold (Clontech) using the Yeastmaker™ Yeast Transformation System 2 (Clontech). The cDNA library of *V. harveyi*-infected shrimp hemocytes used to screen for the interaction with the RBD of A2M was constructed using the Make Your Own “Mate & Plate™” Library system (Clontech). In brief, the hemocytes from five individual shrimp previously infected with 10^6^ CFU of *V. harveyi* were harvested at 6 or 48 h post-infection. The total RNA was extracted and cDNA was synthesized as described previously [32]. The library was constructed according to the manufacturer's protocol. Yeast mating and selection of positive transformants were carried out according to the Matchmaker™ Gold Y2H System user manual (Clontech). The prey plasmids were rescued as previously reported [33] and later confirmed the interaction by the co-transformation assay, as described in the instruction manual. The prey plasmid which truly interacts with the RBD of A2M was subjected to DNA sequencing. The obtained sequences were searched against the GenBank database using the BlastX program.

**Table 1:** DNA sequences of the primers used in experiments.

Primers	Primer sequences (5′-3′)
**Yeast two-hybrid**	
*Pm*A2M-F	5**′**-GCCGATCATATGCTTGTCCACGAATTCACC-3**′**
*Pm*A2M-R	5**′**-ATCGAGGATCCTTTTGCTGCCGTCCAC-3**′**
**Protein production**	
*Pm*A2M-F	5**′**-CGCGGCCATATGCTTGTCCACGAATTCACC-3**′**
*Pm*A2M-R	5**′**-CATATGCTCGAGTTTGCTGCCGTCCACCTCGTA-3**′**
**Gene silencing**	
*Pm*A2M-F	5**′**-CCATGGAGGGGCAGGGATGC-3**′**
*Pm*A2M-R	5**′**-ATCGCACCCTTCGAGGTACG-3**′**
T7*Pm*A2M-F	5**′**-GGATCCTAATACGACTCACTATAGGCCATGGAGGGGCAGGGATGC-3**′**
T7*Pm*A2M-R	5**′**-GGATCCTAATACGACTCACTATAGGATCGCACCCTTCGAGGTACG-3**′**
**Semiquantitative RT-PCR analysis**	
*Pm*A2M-F	5**′**-CCATGGAGGGGCAGGGATGC-3**′**
*Pm*A2M-R	5**′**-ATCGCACCCTTCGAGGTACG-3**′**
*β*-actin-F	5**′**-GCTTGCTGATCCACATCTGCT-3**′**
*β*-actin-R	5**′**-ATCACCATCGGCAACGAGA-3**′**

### Production of recombinant *Pm*RBD-A2M and anti-*Pm*RBD-A2M Abs

The forward and reverse primers (Table 1), with 5′ flanking *Nde*I and *Xho*I sequences (underlined), respectively, were designed to amplify the RBD of A2M from the pGBKT7-RBD-A2M plasmid using the Advantage®2 Polymerase Mix (Clontech). The purified PCR product was digested with *Nde*I and *Xho*I, and then ligated to the pET-22b (+) expression vector (Novagen) cut with the same restriction enzymes. The obtained recombinant plasmid (pET-RBD-A2M) was then transformed into *E. coli* strain BL21(DE3). The transformed *E. coli* was cultured and the protein expression was induced by addition of 1 mM IPTG (Isopropyl β-D-1-thiogalactopyranoside). At 4 h after IPTG induction, the cells were harvested, resuspended in phosphate buffered saline pH 7.4 (PBS) and then sonicated to break the cells. The inclusion bodies were collected, solubilized with 50 mM sodium carbonate pH 10.3, and dialyzed against 20 mM sodium phosphate pH 8.0 overnight at 4°C to refold the protein. The protein was purified using a Ni-Sepharose™ 6 Fast Flow affinity column (GE Healthcare), in accordance with the manufacture's manual. SDS-PAGE and western blotting using the anti-HIS antibody (GE Healthcare) were performed to analyze the purity of the recombinant protein. The polyclonal Abs against the recombinant *Pm*-RBD-A2M were commercially raised in rabbits and mice at the Biomedical Technology Research Unit, Chiang Mai University, Thailand.

### Localization of *Pm*A2M protein in shrimp hemocytes by immunofluorescence

To examine the *Pm*A2M protein localized in hemocytes, hemolymph was collected from three individual shrimp and then pooled. Hemocytes were isolated by centrifugation (800× *g*; 10 min; 4°C), with the hemocyte pellet being immediately fixed in 4% (w/v) paraformaldehyde in PBS and kept at 4°C until use.

Hemocytes (1×10^5^ cells/ml) were attached onto polylysine-coated microscope slides by cytospin centrifugation. Based on immunofluorescence staining procedure previously described [34], *Pm*A2M was probed using purified rabbit anti-*Pm*-RBD-A2M polyclonal IgG Ab diluted 1∶500 in PBSF (PBS plus 1% (v/v) FBS) followed by a 1∶500 dilution of Alexa Fluor® 488 goat anti-rabbit IgG Ab (Invitrogen) in PBSF. Hemocyte nuclei were finally stained. The microscope slides were kept in the dark at 4°C until they were observed under a confocal fluorescence microscope.

### Activation of shrimp blood clotting and preparation of a clot matrix

Two hundred µl of shrimp hemolymph was collected from a ventral haemoceol using a sterile syringe with a 23G needle containing 50 µl of anticoagulant (10% (w/v) sodium citrate). To activate the blood coagulation, 5 µl of 1 M CaCl_2_ was added to the plasma, gently mixed and incubated at ambient temperature for 5 min then stopping the clotting reaction by the addition of 25 µl of 0.5 M EDTA. The clot matrix was fixed onto a polylysine-coated microscope slide using the hanging drop technique, as previously pointed out [35].

### Co-localization of *Pm*A2M and clottable proteins (CPs) on the blood clot by *in vitro* immunohistochemical staining

After preparing clot matrices on the polylysine-coated slides, the samples were air-dried at 37°C in an oven. For immunohistochemical staining [34], the samples were incubated with the primary Abs (purified mouse anti-*Pm*-RBD-A2M polyclonal IgG Ab at 1∶150 dilution in PBSF and the purified rabbit anti-CP polyclonal IgG Ab at 1∶250 dilution in PBSF), simultaneously introduced to the samples, and subsequently probed with 1∶500 dilution of Alexa Fluor®568 goat anti-mouse (Invitrogen) and Alexa Fluor®647 goat anti-rabbit Abs (Invitrogen) in PBSF, respectively. The samples were stored in the dark at 4°C until observed under a confocal fluorescence microscope.

### RNAi-mediated *Pm*A2M gene silencing and semi-quantitative determination of *Pm*A2M transcript levels

Clone number HC-N-N01-1034 from contig 711 of the *P. monodon* EST database (http://pmonodon.biotec.or.th) was found to be similar to the RBD of *Pm*A2M and so was used as the template for *Pm*A2M dsRNA production. Two sets of primers specific to the RBD of *Pm*A2M were designed as shown in Table 1, plus the same primers but 5′ flanked by the T7 promoter sequence (underlined) to form T7*Pm*A2M-F and T7*Pm*A2M-R. Otherwise, dsRNA of GFP, the negative control, was prepared as previously described [36]. The two PCR products were separately amplified using those primer pairs with the following conditions; 94°C for 5 min followed by 30 cycles of 94°C for 30 s, 57°C for 30 s and 72°C for 30 s, and then a final 72°C for 10 min.

Experimental shrimp, each of approximately 1–3 g body weight, were divided into three groups. The first control group was injected with 50 µl of sterile saline solution (SSS) alone. The second and third groups (control and knockdown groups) were injected with 10 µg of either GFP-dsRNA or A2M-dsRNA in 50 µl of SSS, respectively. The injection was repeated as above after 24 h, and the hemolymph was collected from each shrimp in all three groups after a further 24 h (48 h after the first injection). The hemocytes were isolated by centrifugation. Total RNA was extracted and used for cDNA synthesis [32]. Using semi-quantitative RT-PCR and 1.5% (w/v) agarose-TBE gel electrophoresis, the *Pm*A2M gene knockdown level was assessed by comparison between the transcript levels of the *Pm*A2M gene relative to that of the internal housekeeping control gene, *β*-actin (Forward and reverse primers, shown in Table 1). The band densitometry software (GeneTools) was carried out to quantify the band intensity.

### Clot observation under the scanning electron microscope (SEM)

Scanning electron microscopy (SEM) was used to observe the effect of A2M-dsRNA-mediated knockdown on the physiological changes of the clot in the presence of *V. harveyi*. As previously reported [35], [37], the bacterial suspension in PBS was prepared and 1.5 µl of the suspension was added into the plasma from the GFP-dsRNA and A2M-dsRNA treated groups. Blood coagulation was activated as described above and the hanging drop technique [35] was then performed.

To prepare the samples for SEM analysis, the surface of the partially clotted plasma was attached upside down to a new small glass slide avoiding making contact between the two slides. The samples were prepared as described previously [34] and examined under a scanning electron microscope.

### Invading bacteria count in the hemolymph of the *Pm*A2M depleted (dsRNA-mediated knockdown) shrimp

1.5×10^5^ CFU/ml of freshly prepared *V. harveyi* suspended in 50 µl of SSS was intramuscularly injected into the GFP-dsRNA and A2M-dsRNA knockdown groups. Three independent individuals were evaluated per group, and for each shrimp 100 µl of hemolymph was collected using a sterile syringe without any anticoagulant solution- at 5, 15 or 30 min after injection. Ten µl from each collected hemolymph sample was immediately dotted on TCBS (thiosulfate–citrate–bile–sucrose) agar plates, a *Vibrio* species selective medium, with five replicates performed per sample. After air-drying the dotted hemolymph, the plates were then incubated upside down at 30°C overnight. The number of invading bacteria was counted by a total plate count method, as previously pointed out [38].

## Results

### Carboxyl terminus of transglutaminase type II (*Pm*STG II) identified as an interacting partner of the receptor binding domain (RBD) of *Pm*A2M

Previously, proteomic analysis revealed the significant down-regulation of shrimp A2M protein levels in response to *V. harveyi* infection [28], [29]. It is evident that the RBD of A2M plays necessary roles in subsequent clearance of the protease-reacted A2M from the blood circulation. Thus, the protein partners that likely interact with the RBD of *Pm*A2M were identified using the Y2H system to further investigate the potential role of A2M in shrimp immunity. A DNA fragment encoding the RBD of *Pm*A2M was initially cloned into pGBKT7. Before performing the Y2H assay, the recombinant *Pm*A2M in the pGBKT7 backbone was tested for autoactivation of yeast reporter genes and toxicity to host yeast cells. No activation of the reporter genes or differences in yeast cell sizes were observed between the control and experimental groups (data not shown).

After the screening, six independent positive clones (SB1–4, PB1 and PB2) were discovered. The prey plasmids from these clones were isolated and subjected to DNA sequencing. Four of the positive clones (SB1–4) were identified by sequence identity as being the carboxyl-terminus of transglutaminase type II, *Pm*STG II (accession number AAV49005.1). The other two clones, PB1 and PB2, were identified by sequence similarity to be the homologs of the voltage-dependent anion-selective channel isoform 1 from *Tribolium castaneum* (accession number XP_967480.1) and the 40S ribosomal protein S20 from *Rimicaris exoculata* (accession number ACR78697.1), respectively.

**Figure 1:**
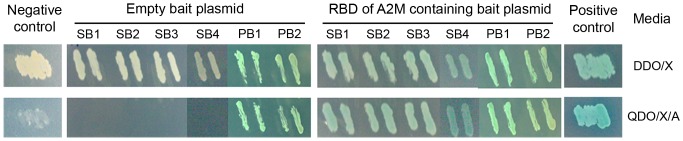
Y2H based co-transformation assay to confirm positive prey proteins. The RBD of A2M was used as the bait protein. The prey plasmids encoding for the carboxyl-terminus of the *P. monodon* transglutaminase type II (*Pm*STG II) (SB1, SB2, SB3 and SB4), and the homologs of the *T. castaneum* voltage-dependent anion-selective channel isoform 1 (PB1) and *R. exoculata* 40S ribosomal protein S20 (PB2), were co-transformed into the *S. cerevisiae* strain Gold with either an empty bait plasmid or a bait plasmid containing the RBD of A2M. The yeast transformants grown on selective media were restreaked onto DDO/X media (double drop-out media lacking leucine and tryptophan but supplemented with X-α-Gal) and QDO/X/A (quadruple drop-out media lacking adenine, histidine, leucine and tryptophan but supplemented with X-α-Gal and Aureobasidin A, a high stringency medium) and evaluated for growth. The interaction between murine p53 and the SV40 large T antigen served as a positive control, whereas that between the murine p53 and lamin was used as the negative control.

These six purified prey plasmids were then ascertained if they authentically interact with the bait by the co-transformation assay. Like the positive control, all the transformants containing the SB1–4 and PB1–2 prey and bait plasmids seemed to grow well and appeared as blue colonies on both selective media, as a result of reporter gene activations. Correspondingly, no growth was observed in the SB1–4 prey and empty bait plasmids co-transformed yeast cells on the high stringency media, similar to the negative control (Fig. 1). On the other hand, the PB1 and PB2 prey and empty bait plasmids containing transformants grew on the high stringency media, suggesting that the PB1 and PB2 clones were false positives (Fig. 1). Altogether, the results support that the carboxyl-terminal region of the *Pm*STG II protein(s) interact(s) with the RBD of *Pm*A2M.

### *Pm*A2M is mainly contained in semigranular hemocytes

**Figure 2:**
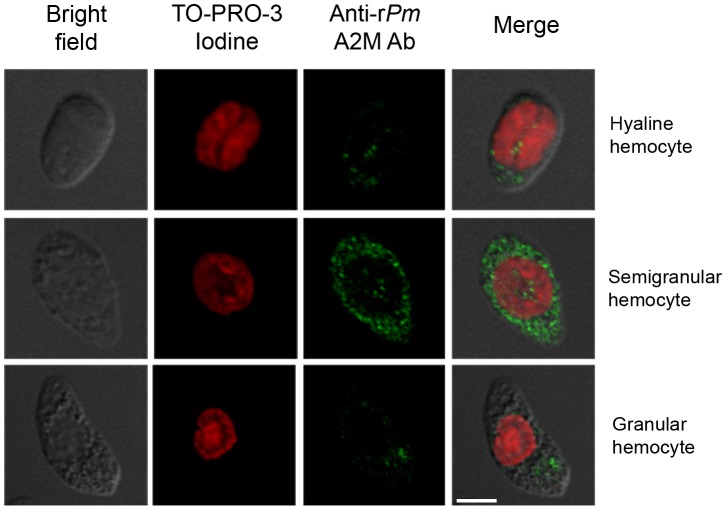
Confocal microscope analysis of A2M protein content in shrimp hemocytes using immunohistochemical staining. Three different types of the hemocytes consisting of hyaline, semigranular, and granular hemocytes are indicated and pooled hemocytes from three normal shrimp (1×10^5^ cells/ml) are shown. The A2M proteins and hemocytic nuclei are visualized as green (Alexa Fluor® 488) and red (TO-PRO-3 Iodine) colors, respectively (colocalization of the nucleus and A2M is not seen in the merged image). The scale bar corresponds to 5 µm. Images shown are representative of at least 3 such fields of view per sample.

According to our proteomic study concerning responsive proteins in hemocytes of *V. harveyi* infected *P. monodon* [28] suggesting that hemocytes are one of the principle sources of *Pm*A2M involving in bacterial defense, the localization of *Pm*A2M was then determined in three main types of shrimp hemocytes (hyaline, semigranular, and granular hemocytes). Immunofluorescent staining of the *Pm*A2M was conducted to assess the amount of *Pm*A2M protein in hemocytes. The hemocyte nuclei were visualized by staining with TO-PRO-3 iodine (red color in Fig. 2), while anti-r*Pm*A2M Ab and Alexa Fluor® 488 conjugated second Ab were applied to detect the subcellular *Pm*A2M protein (green color in Fig. 2).

Fig. 2 demonstrated the aspect of shrimp hemocytes which are mainly classified into three types; hyaline (agranular), semigranular (small-granular), and granular (large-granular) hemocytes, based on the existence or relative size of granules [39]. The strongest immunostaining for *Pm*A2M was noticed in the semigranular hemocytes, and was contained within the secretory granules of those cells. There were also a few immunostaining granules within the granular hemocytes, but positive *Pm*A2M staining hyaline cells were rarely observed (Fig. 2). The result indicated that the semigranular hemocyte is a major component among other types which might play crucial roles in *Pm*A2M regulation against the bacterial infection.

### The incorporation of *Pm*A2M in shrimp clots

Since the Y2H screening revealed that *Pm*A2M was able to interact with *Pm*STG II, a key enzyme that plays a crucial role in the blood coagulation system [40], [41], it is possible that *Pm*A2M is one of *Pm*STG II's interacting partners and can be associated with the clotting system. Immunofluorescence using confocal microscopy was then conducted to examine the presence of *Pm*A2M in the shrimp clots. CPs, known as a backbone of the fibril clot, were investigated for colocalization with *Pm*A2M to demonstrate the location of hemolymph clot matrix.

**Figure 3:**
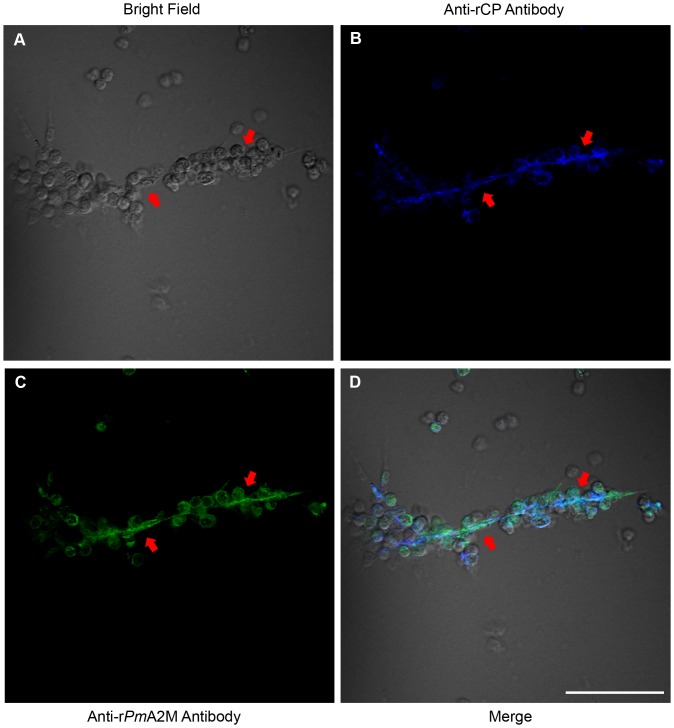
Co-localization of A2M and CPs in hemocytes and extracellular fibrils of blood clots. The immunocytology and confocal microscopy were performed. Anti-r*Pm*A2M and anti-rCP Abs were visualized by Alexa Fluor® 568 and Alexa Fluor® 647 conjugated secondary Abs and show up as green and blue colors for A2M and CP, respectively, or cyan for colocalized in the merged field. Red arrowheads demonstrate fibril clots and the scale bar corresponds to 50 µm. Images shown are representative of at least 3 such fields of view per sample.

Using the hanging drop technique with CaCl_2_ activated blood clotting, *Pm*A2M and CPs were detected by anti-r*Pm*A2M mouse IgG and anti-rCP rabbit IgG Abs, respectively, followed by Alexa Fluor® 568 conjugated goat anti-mouse and Alexa Fluor® 647 conjugated goat anti-rabbit IgG Abs, respectively. As can be seen in the bright field, in the activated clotting condition, plasma clot strands developed allowing the association of many hemocytes onto the clot fibrils (Fig. 3A; red arrows). CPs were found to be specifically dispersed on the plasma clotting fibril strands (blue color in Fig. 3B). Interestingly, *Pm*A2M was also localized on the clot strands and the associated hemocyte cells (green color in Fig. 3C), such that the CPs and *Pm*A2M were colocalized over much of their distribution in the extracellular fibrils (cyan color in Fig. 3D). However, the two components are not totally colocalized, with significant areas of both fibrils and hemocytes being stained for either CP and not *Pm*A2M, or *Pm*A2M and not CP. Nevertheless, overall *Pm*A2M was clearly incorporated onto the extracellular blood clot matrix.

### Partially depleted *Pm*A2M transcript levels result in the escape of *V. harveyi* from the clot

**Figure 4:**
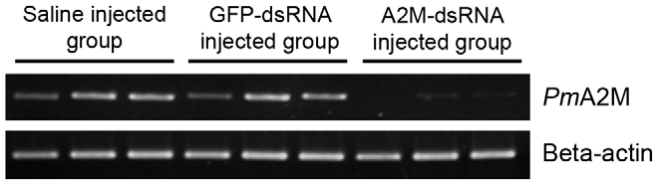
Determination of the A2M transcript in the shrimp hemocytes after *in vivo* RNAi-mediated gene silencing. Three groups of experimental shrimp (1–3 g body weight) were injected with SSS alone or with SSS containing either GFP-dsRNA or A2M-dsRNA in a final concentration of 10 µg/g body weight. Each group contains three individuals and each lane represents an individual shrimp. The mRNA expression level of A2M was assessed by semi-quantitative RT-PCR using A2M gene specific primers and normalized relative to that for the *β*-actin gene, which served as the internal control.

To elucidate the biological function of *Pm*A2M, dsRNA interference mediated gene silencing was performed in order to suppress *Pm*A2M transcript (and so assumed protein) expression levels in *P. monodon* shrimp. Ten µg of dsRNA specific to the RBD of *Pm*A2M (A2M-dsRNA) in 50 µl of SSS was injected into each experimental shrimp twice with a 24 h interval between them. Control shrimp (two groups) were likewise injected but with either SSS alone or SSS containing dsRNA against GFP (GFP-dsRNA). A significant (∼94%) reduction in the *Pm*A2M transcript level was observed in the hemocytes from the dsRNA-A2M knockdown shrimp at 24 h after the second injection, as determined by semi-quantitative RT-PCR, but no change was noted in the dsRNA-GFP or SSS control groups (Fig. 4). The *Pm*A2M transcript level recovered to normal levels by day 5 (data not shown).

**Figure 5:**
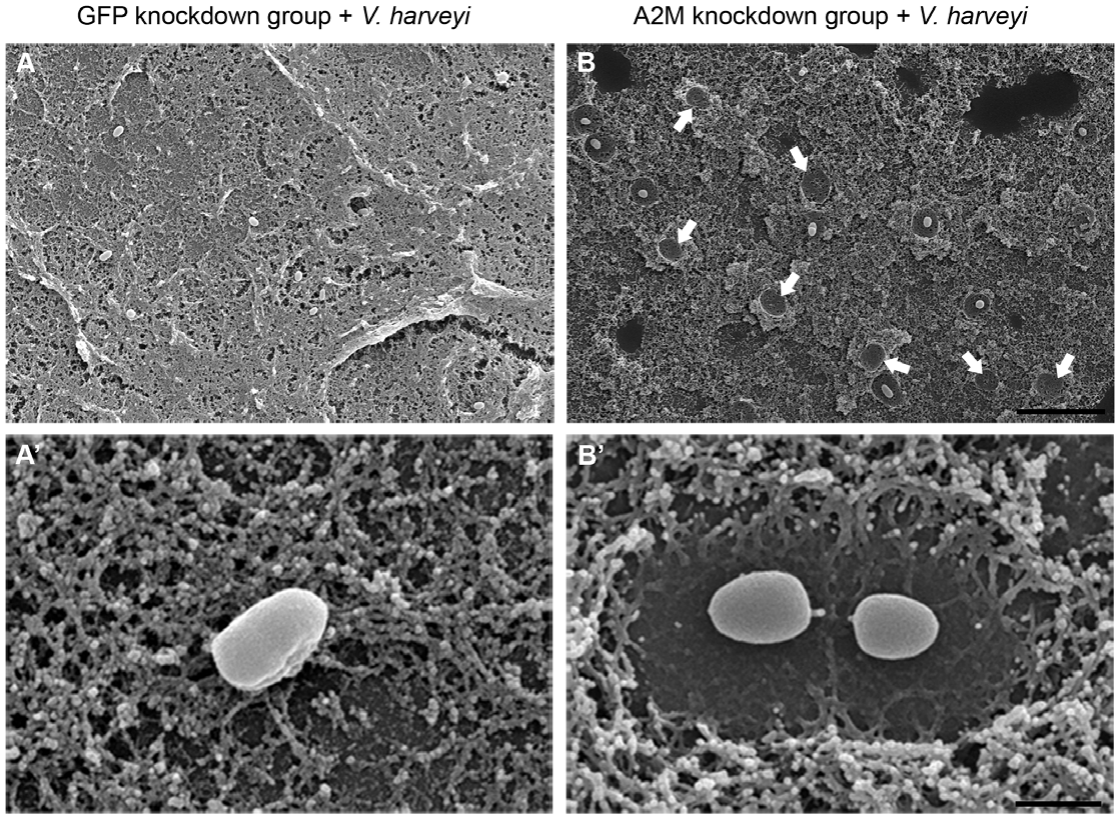
Observation of GFP-dsRNA and A2M-dsRNA treated clots in the presence of *V. harveyi*. SEM was used at an accelerating voltage at 20 kV and a magnification of 2,000× (**A**, **B**) or 20,000× (**A′**, **B′**) were applied. Blood clots were formed by the hanging drop technique in the presence of *V. harveyi* from the hemolymph of (**A**, **A′**) GFP-dsRNA or (**B**, **B′**) A2M-dsRNA knockdown shrimp. Arrowheads demonstrate the fibrinolysis zones where the bacteria have escaped from the clot. Scale bars correspond to 10 µm in the upper sub-figures and 1 µm in the lower sub-figures. Images shown are representative of at least 3 such fields of view per sample.

The effect of this significantly reduced *Pm*A2M transcript levels in the A2M gene silenced shrimp on the ability to entrap *V. harveyi* in the plasma clot was evaluated. To this end, hemolymph obtained from GFP-dsRNA control and A2M-dsRNA knockdown shrimp was mixed with *V. harveyi* and clotting was induced. Samples were then prepared, CO_2_ critical dried, and examined by SEM. When *V. harveyi* was present during the clotting, the bacteria became entrapped in the extracellular blood clots of the control group (Fig. 5A). There were some fibrils attached to the bacteria, presumably immobilizing them in the clot, and the bacteria remained in close association with the clot matrix (Fig. 5A′). Strikingly, the blood clot matrix observed in the A2M-dsRNA treated shrimp showed that the remaining bacteria were surrounded by fibrinolysis zones (Fig. 5B′), indicating the susceptibility of A2M-depleted clots to proteolysis, presumably from proteases released from the bacterial cells [42]. Moreover, some of the invading bacteria had apparently escaped from the clot matrix, as can be seen in some regions (Fig. 5B; arrows), suggesting that *Pm*A2M bound to the clots prevented bacterial escape by inhibiting the bacterial protease-mediated fibrinolysis.

### Systemic dissemination of *V. harveyi* in the circulating hemolymph of *Pm*A2M gene silenced shrimp

**Figure 6:**
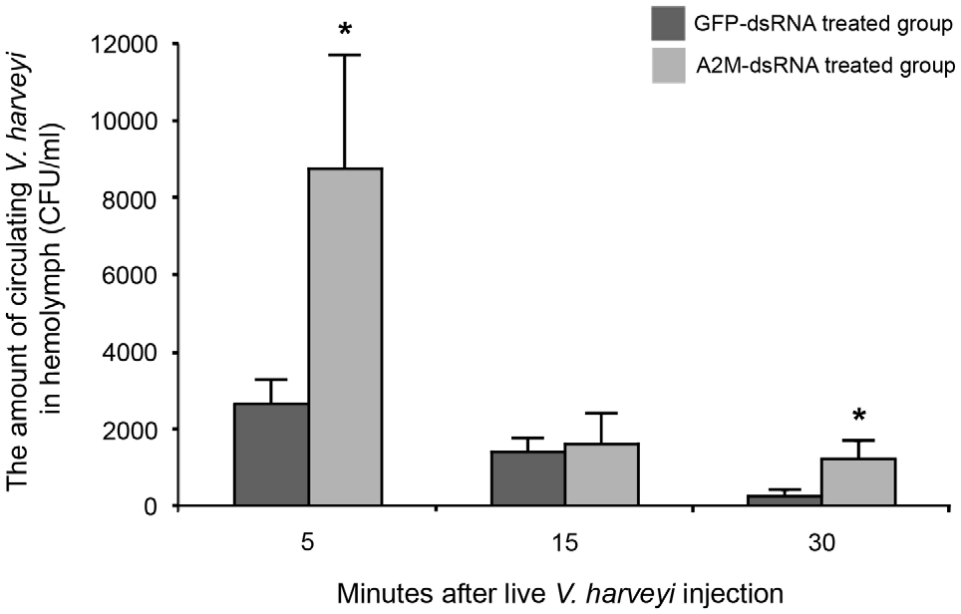
The evaluation of invading *V. harveyi* levels in the hemolymph. Hemolymph was collected from GFP and A2M knockdown shrimp at 5, 15 and 30 min after injection with freshly prepared *V. harveyi* (1.5×10^5^ CFU/ml) and then plated onto *Vibrio* sp. selective agar plates (TCBS) to examine the number of viable bacteria. The amount of circulating bacteria in hemolymph of the GFP knockdown group was used as control. Five trials were done in each group. The data, as CFU/ml, are shown as the mean ± S.D. * represents a significant difference between the two shrimp groups (*p*<0.05; Independent Sample T-test).

The number of disseminated *V. harveyi* in the shrimp hemolymph was counted in order to support the above finding of the apparent escape of bacteria from clots in the A2M knockdown shrimp. The hemolymph from GFP-dsRNA and A2M-dsRNA treated shrimp was collected at 5, 15 and 30 min after injection of live *V. harveyi*. At 5 min, the number of bacteria in the A2M-dsRNA treated group was significantly (3.3-fold) higher than that in the control group. The invading bacteria were, nevertheless, cleared out from the circulatory system in the control group by 30 min, and reduced some four-fold to <2000 CFU/ml in the A2M depleted shrimp by 15 min (Fig. 6). Additionally, there were a few of the viable bacteria detected in hemolymph of both groups at 6 h and 24 h (no significant difference) and these viable bacterial numbers kept slightly decreasing over the time (data not shown). Since the differences in the bacterial number were only seen at the most early time points of infection (5 min), it suggests that the bacterial entrapment within the plasma clots would represent the early immune mechanisms in response to the bacterial infection.

The results demonstrated the release of *V. harveyi* cells from the A2M-depleted blood clots into the circulatory system, whilst in the control shrimp, most bacteria were not able to enter the circulation system and so were presumably entrapped in the clot. This supported the important function of A2M in shrimp innate immunity by preventing the escape of bacteria from blood clots.

## Discussion

In order to better understand the innate immunity of crustaceans, shrimp immune responses to several pathogens have been elucidated from a variety of proteomic, genomic, molecular genetical and biochemical approaches. From the immune responsive proteins identified and studied to date by these methods, A2M expression levels were found to be highly altered after pathogen infection in several shrimp species including *P. monodon*. Even though the changes in A2M expression levels in the major immune tissues were typically observed when challenged with viruses, such as white spot syndrome and yellow head viruses [43]–[46], a substantial change in both the transcript and protein levels of A2M was noticed after bacterial challenge, and particularly after *V. harveyi* infection [25], [28], [29]. Moreover, Gollas-Galvan *et al.* [20] recently revealed that the A2M from *P. vannamei* exhibits a broad range of protease inhibitory activities to various proteases, including members of the elastase and aminopeptidase families that include known pathogen-aiding bacterial proteases. Hence, changes in shrimp A2M expression levels might be a host defense mechanism against bacterial proteases, possibly to neutralize the severity of the bacterial infection or to prevent the bacterial dissemination throughout the shrimp hemoceol. The biological pathway to inhibit the action of bacterial proteases used by A2M in shrimp, nevertheless, remains elusive.

To unveil the defense action of *Pm*A2M against bacteria, potential molecules that interact with *Pm*A2M was investigated using the Y2H screening assay. The RBD of *Pm*A2M was found to interact with the carboxyl-terminal domain of *Pm*STG II. Currently, at least three members of the STG enzyme family have been identified in *P. monodon*, which are *Pm*STG I (AY074924) [47] and two members of *Pm*STG II (AY771615; AF469484) [41]. However, the amino acid sequences of the C-terminus region of both known *Pm*STG II proteins are congruent and therefore further experiments are required to investigate which *Pm*STG II isoform is interacting with A2M, or if both isoforms are involved. Since both *Pm*STG IIs were previously determined to be key enzymes involved in the blood coagulation system [40], [41], the binding between *Pm*A2M and *Pm*STG II suggests a possible role for *Pm*A2M related to the hemolymph clotting. In accordance with the protein polymerization activity of STGs, which catalyses an acyl transfer reaction between lysine residues and glutamine residues, four conserved lysine residues (K1324, K1387, K1421 and K1425) were discovered in the RBD of *Pm*A2M [22]. In addition, the A2M of the crayfish, *P. leniusculus*, can act as a substrate of transglutaminase and exposes free lysine and glutamine residues as does the A2M of the American horseshoe crab, *Limulus polyphemus*, which decorates on the clot in the presence of Ca^2+^ [18], [42], [48]. These data suggest that *Pm*A2M might also be a substrate of the transglutaminase enzyme for cross-linking to other proteins serving roles in the plasma coagulation. Although there have been a number of reports on mammalian A2M revealing that the RBD can bind to the low-density lipoprotein receptor-related protein A2M receptor (LRP-A2MR) mediating the clearance of inhibitor–protease complexes from the blood circulation and the LRP receptor-associated protein (RAP) mediating a signal regulation [49], they have no report in invertebrates including shrimp. It can be proposed that the KPTVK motif which is responsible for receptor binding [50] is not present in the RBD of *Pm*A2M; therefore, shrimp might possess other pathways for the protease clearance.

In invertebrates, the hemolymph coagulation process commonly occurs in plasma with the cooperation of a variety of hemocyte derived molecules but without the direct involvement of the hemocytes [51]. Blood coagulation in shrimp has been proposed to be similar to that in the crayfish, *P. leniusculus* [40], where under an external stimulus, such as LPS, STG is released from the hyaline hemocytes into the plasma to initiate the blood clotting by cross-linking the CPs, which are abundant proteins in the hemolymph that are mainly produced in the gill, central nervous system and the lymphoid organ [52], [53]. The result of this report here demonstrated that *Pm*A2M is contained in semigranular hemocytes (Fig. 2) and possibly releases into the hemolymph via degranulation. Moreover, an increase of A2M protein was observed in the plasma upon *V. harveyi* infection (data not shown). We assume that the secreted A2M participates in several host immune defenses occurring in plasma including the hemolymph clotting process. This might be a reason why the A2M protein content in shrimp hemocytes was substantially decreased upon the *V. harveyi* infection, observed in our proteomic study [28]. As previously studied in the horseshoe crab, *Limulus polyphemus* [54], the exocytosis of granules packed in the blood cells was activated by endotoxin to subsequently release A2M into the plasma. This is the first report in shrimp that demonstrates the direct involvement of A2M in blood coagulation and that A2M is incorporated into the clots where it potentially serves as one of the major backbones of the clot matrix along with the CPs (Fig. 3).

To gain more insight into the biological role of *Pm*A2M in the blood clotting, we observed the effect of *Pm*A2M gene silencing on the entrapment of bacteria in the clots by SEM. With the partial RNAi-mediated knockdown of *Pm*A2M gene transcript expression, the *Pm*A2M depleted plasma clots were notably ineffective at sequestering *V. harveyi*, resulting in the higher systemic dissemination of the bacteria into the circulatory system (Figs. 5 and 6). Nevertheless, the invading bacteria were rapidly cleared out from the blood circulation in both control and A2M gene silenced shrimp within half an hour. It is not so surprised because an array of host immune responses actively functions against invading pathogens and is doubtlessly required for the pathogen clearance. This is the first evidence suggesting that *Pm*A2M plays a critical role in the blood clotting system by preventing fibrinolysis of clots from *V. harveyi* proteases, and so in restraining the protease-mediated bacterial escape.

The physical entrapment mechanism of A2M has been proposed to be comprised of three main steps. Firstly, in the protease recognition step, a protease directly attacks some susceptible peptide bonds in the bait region followed by the compaction of the A2M molecule to entrap the protease. Secondly, in the capture step, the thiol group is cleaved and generates a reactive glutamyl group which can covalently link to the protease. Lastly, as a result of this conformation change, the RBD of A2M is exposed on the outside of the molecule and the domain is recognized by its receptor for subsequent entry to the endocytotic clearance pathway [49], [55]. Thus, it seems that only a protease reacted form of A2M, where the RBD is uncovered, will be biologically processed whilst its unreacted form is still free in both the plasma and hemocytes. The fibril bound A2M in the American horseshoe crab had already functioned and appeared as a reacted form [48], whereas, *Pm*A2M on the shrimp clots is able to inhibit the proteolysis activity of, presumably, proteases secreted from the invading bacteria [42] (Figs. 5A and 5A′), implying that the exposure of the RBD of *Pm*A2M occurs when linked on the clots and before entrapping any proteases. It should be noted that the reorganization of the molecular structure of *Pm*A2M is probably distinct between the different isoforms and so this requires further investigation for clarification. Altogether, our findings clearly indicate a vital role for *Pm*A2M in the blood coagulation system, the most efficient primitive immune response, against pathogen attack.

## References

[1] Dorsch M, Lane D, Stackebrandt E (1992) Towards a phylogeny of the genus *Vibrio* based on 16S rRNA sequences. Int J Syst Bacteriol 42: 58–63. 1371064 10.1099/00207713-42-1-58

[2] Jiravanichpaisal P, Miyazaki T, Limsuwan C (1994) Histopathology, biochemistry, and pathogenicity of *Vibrio harveyi* infecting black tiger prawn *Penaeus monodon* . J Aquat Anim Health 6: 27–35.

[3] Austin B, Zhang XH (2006) *Vibrio harveyi*: a significant pathogen of marine vertebrates and invertebrates. Lett Appl Microbiol 43: 119–124. 16869892 10.1111/j.1472-765X.2006.01989.x

[4] Armstrong PB (2006) Proteases and protease inhibitors: a balance of activities in host-pathogen interaction. Immunobiology 211: 263–281. 16697919 10.1016/j.imbio.2006.01.002

[5] Fukasawa S, Nakamura K, Miyahira M, Kurata M (1988) Some properties of two proteinases from a luminous bacterium, *Vibrio harveyi* strain FLN-108. Agric Biol Chem 52: 3009–3014.

[6] Fukasawa S, Nakamura K, Kamii A, Ohyama Y, Osumi M (1988) Purification and properties of a proteinase from a marine luminous bacterium, *Vibrio harveyi* strain FLA-11. Agric Biol Chem 52: 435–441.

[7] Liu PC, Lee KK, Tu CC, Chen SN (1997) Purification and characterization of a cysteine protease produced by pathogenic luminous *Vibrio harveyi* . Curr Microbiol 35: 32–39. 9175557 10.1007/s002849900207

[8] Liu PC, Lee KK (1999) Cysteine protease is a major exotoxin of pathogenic luminous *Vibrio harveyi* in the tiger prawn, *Penaeus monodon* . Lett Appl Microbiol 28: 428–430. 10389258 10.1046/j.1365-2672.1999.00555.x

[9] Montero AB, Austin B (1999) Characterization of extracellular products from an isolate of *Vibrio harveyi* recovered from diseased post-larval *Penaeus vannamei* (Bonne). J Fish Dis 22: 377–386.

[10] Harris LJ, Owens L (1999) Production of exotoxins by two luminous *Vibrio harveyi* strains known to be primary pathogens of *Penaeus monodon* larvae. Dis Aquat Organ 38: 11–22.

[11] Laskowski M Jr, Kato I (1980) Protein inhibitors of proteinases. Ann Rev Biochem 49: 593–626. 6996568 10.1146/annurev.bi.49.070180.003113

[12] Armstrong PB, Quigley JP (1999) α_2_-macroglobulin: an evolutionarily conserved arm of the innate immune system. Dev Comp Immunol 23: 375–390. 10426429 10.1016/s0145-305x(99)00018-x

[13] Kanost MR (1999) Serine proteinase inhibitors in arthropod immunity. Dev Comp Immunol 23: 291–301. 10426423 10.1016/s0145-305x(99)00012-9

[14] Armstrong PB, Melchior R, Quigley JP (1996) Humoral immunity in long-lived arthropods. J Insect Physiol 42: 53–64.

[15] Buresova V, Hajdusek O, Franta Z, Sojka D, Kopacek P (2009) IrAM-An α_2_-macroglobulin from the hard tick *Ixodes ricinus*: Characterization and function in phagocytosis of a potential pathogen *Chryseobacterium indologenes* . Dev Comp Immunol 33: 489–498. 18948134 10.1016/j.dci.2008.09.011

[16] Levashina EA, Moita LF, Blandin S, Vriend G, Lagueux M, et al (2001) Conserved role of a complement-like protein in phagocytosis revealed by dsRNA knockout in cultured cells of the mosquito, *Anopheles gambiae* . Cell 104: 709–718. 11257225 10.1016/s0092-8674(01)00267-7

[17] Aspán A, Hall M, Söderhäll K (1990) The effect of endogeneous proteinase inhibitors on the prophenoloxidase activating enzyme, a serine proteinase from crayfish haemocytes. Insect Biochem 20: 485–492.

[18] Hall M, Söderhäll K (1994) Crayfish α-macroglobulin as a substrate for transglutaminases. Comp Biochem Phys B 108: 65–72.

[19] Armstrong PB, Levin J, Quigley JP (1984) Role of endogenous proteinase inhibitors in the regulation of the blood clotting system of the horseshoe crab, *Limulus polyphemus* . Thromb Haemostas 52: 117–120. 6084318

[20] Gollas-Galván T, Sotelo-Mundo RR, Yepiz-Plascencia G, Vargas-Requena C, Vargas-Albores F (2003) Purification and characterization of α_2_-macroglobulin from the white shrimp (*Penaeus vannamei*). Comp Biochem Phys C 134: 431–438. 10.1016/s1532-0456(03)00002-4 12727292

[21] Rattanachai A, Hirono I, Ohira T, Takahashi Y, Aoki T (2004) Molecular cloning and expression analysis of α_2_-macroglobulin in the kuruma shrimp, *Marsupenaeus japonicus* . Fish Shellfish Immunol 16: 599–611. 15110334 10.1016/j.fsi.2003.09.011

[22] Lin YC, Vaseeharan B, Ko CF, Chiou TT, Chen JC (2007) Molecular cloning and characterisation of a proteinase inhibitor, alpha 2-macroglobulin (α2-M) from the haemocytes of tiger shrimp *Penaeus monodon* . Mol Immunol 44: 1065–1074. 16982096 10.1016/j.molimm.2006.08.002

[23] Lin YC, Vaseeharan B, Chen JC (2008) Molecular cloning and phylogenetic analysis on α2-macroglobulin (α2-M) of white shrimp *Litopenaeus vannamei* . Dev Comp Immunol 32: 317–329. 17706773 10.1016/j.dci.2007.07.002

[24] Ho PY, Cheng CH, Cheng W (2009) Identification and cloning of the α2-macroglobulin of giant freshwater prawn *Macrobrachium rosenbergii* and its expression in relation with the molt stage and bacteria injection. Fish Shellfish Immunol 26: 459–466. 19340942 10.1016/j.fsi.2009.01.007

[25] Ma H, Wang B, Zhang J, Li F, Xiang J (2010) Multiple forms of alpha-2 macroglobulin in shrimp *Fenneropenaeus chinesis* and their transcriptional response to WSSV or *Vibrio* pathogen infection. Dev Comp Immunol 34: 677–684. 20105438 10.1016/j.dci.2010.01.014

[26] Tonganunt M, Phongdara A, Chotigeat W, Fujise K (2005) Identification and characterization of syntenin binding protein in the black tiger shrimp *Penaeus monodon* . J Biotechnol 120: 135–145. 16055222 10.1016/j.jbiotec.2005.06.006

[27] Chotigeat W, Deachamag P, Phongdara A (2007) Identification of a protein binding to the phagocytosis activating protein (PAP) in immunized black tiger shrimp. Aquaculture 271: 112–120.

[28] Somboonwiwat K, Chaikeeratisak V, Wang HC, Lo CF, Tassanakajon A (2010) Proteomic analysis of differentially expressed proteins in *Penaeus monodon* hemocytes after *Vibrio harveyi* infection. Proteome Sci 8: 39. 20626881 10.1186/1477-5956-8-39 PMC2915975

[29] Chaikeeratisak V, Somboonwiwat K, Wang HC, Lo CF, Tassanakajon A (2012) Proteomic analysis of differentially expressed proteins in the lymphoid organ of *Vibrio harveyi*-infected *Penaeus monodon* . Mol Biol Rep 39: 6367–6377. 22302389 10.1007/s11033-012-1458-6

[30] Isakova V, Armstrong PB (2003) Imprisonment in a death-row cell: The fates of microbes entrapped in the *Limulus* blood clot. Biol Bull 205: 203–204. 14583530 10.2307/1543253

[31] Ponprateep S, Somboonwiwat K, Tassanakajon A (2009) Recombinant anti- lipopolysaccharide factor isoform 3 and the prevention of vibriosis in the black tiger shrimp, *Penaeus monodon* . Aquaculture 289: 219–224.

[32] Amparyup P, Sutthangkul J, Charoensapsri W, Tassanakajon A (2012) A pattern recognition protein binds to lipopolysaccharide and *β*-1,3-glucan and activates the shrimp prophenoloxidase system. J Biol Chem 287: 10060–10069. 22235126 10.1074/jbc.M111.294744 PMC3322982

[33] Robzyk K, Kassir Y (1992) A simple and highly efficient procedure for rescuing autonomous plasmids from yeast. Nucleic Acids Res 20: 3790. 1641351 10.1093/nar/20.14.3790 PMC334042

[34] Prapavorarat A, Vatanavicharn T, Söderhäll K, Tassanakajon A (2010) A novel viral responsive protein is involved in hemocyte homeostasis in the black tiger shrimp, *Penaeus monodon* . J Biol Chem 285: 21467–21477. 20444692 10.1074/jbc.M110.130526 PMC2898424

[35] Bidla G, Lindgren M, Theopold U, Dushay MS (2005) Hemolymph coagulation and phenoloxidase in Drosophila larvae. Dev Comp Immunol 29: 669–679. 15854679 10.1016/j.dci.2004.11.007

[36] Amparyup P, Charoensapsri W, Tassanakajon A (2009) Two prophenoloxidases are important for the survival of *Vibrio harveyi* challenged shrimp *Penaeus monodon* . Dev Comp Immunol 33: 247–256. 18834900 10.1016/j.dci.2008.09.003

[37] Wang Z, Wilhelmsson C, Hyrsl P, Loof TG, Dobes P, et al (2010) Pathogen entrapment by transglutaminase—A conserved early innate immune mechanism. PLoS Pathog 6: e1000763 doi:10.1371/journal.ppat.1000763. 20169185 10.1371/journal.ppat.1000763 PMC2820530

[38] Liu H, Jiravanichpaisal P, Cerenius L, Lee BL, Söderhäll I, et al (2007) Phenoloxidase is an important component of the defense against *Aeromonas hydrophila* infection in a crustacean, *Pacifastacus leniusculus* . J Biol Chem 282: 33593–33598. 17855335 10.1074/jbc.M706113200

[39] Martin GG, Graves BL (1985) Fine structure and classification of shrimp hemocytes. J Morphol 185: 339–348. 10.1002/jmor.1051850306 29976016

[40] Chen MY, Hu KY, Huang CC, Song YL (2005) More than one type of transglutaminase in invertebrates? A second type of transglutaminase is involved in shrimp coagulation. Dev Comp Immunol 29: 1003–1016. 15985293 10.1016/j.dci.2005.03.012

[41] Yeh MS, Kao LR, Huang CJ, Tsai IH (2006) Biochemical characterization and cloning of transglutaminases responsible for hemolymph clotting in *Penaeus monodon* and *Marsupenaeus japonicus* . BBA-Proteins Proteom 1764: 1167–1178. 10.1016/j.bbapap.2006.04.005 16769260

[42] Theopold U, Schmidt O, Söderhäll K, Dushay MS (2004) Coagulation in arthropods: defence, wound closure and healing. Trends Immunol 25: 289–294. 15145318 10.1016/j.it.2004.03.004

[43] Pongsomboon S, Tang S, Boonda S, Aoki T, Hirono I, et al (2011) A cDNA microarray approach for analyzing transcriptional changes in *Penaeus monodon* after infection by pathogens. Fish Shellfish Immunol 30: 439–446. 20971195 10.1016/j.fsi.2010.10.015

[44] Rojtinnakorn J, Hirono I, Itami T, Takahashi Y, Aoki T (2002) Gene expression in haemocytes of kuruma prawn, *Penaeus japonicus*, in response to infection with WSSV by EST approach. Fish Shellfish Immunol 13: 69–83. 12201653 10.1006/fsim.2001.0382

[45] Wang HC, Wang HC, Leu JH, Kou GH, Wang AHJ, et al (2007) Protein expression profiling of the shrimp cellular response to white spot syndrome virus infection. Dev Comp Immunol 31: 672–686. 17188354 10.1016/j.dci.2006.11.001

[46] Bourchookarn A, Havanapan P, Thongboonkerd V, Krittanai C (2008) Proteomic analysis of altered proteins in lymphoid organ of yellow head virus infected *Penaeus monodon* . BBA-Proteins Proteom 1784: 504–511. 10.1016/j.bbapap.2007.12.006 18206130

[47] Huang CC, Sritunyalucksana K, Söderhäll K, Song YL (2004) Molecular cloning and characterization of tiger shrimp (*Penaeus monodon*) transglutaminase. Dev Comp Immunol 28: 279–294. 14698215 10.1016/j.dci.2003.08.005

[48] Armstrong PB, Armstrong MT (2003) The decorated clot: Binding of agents of the innate immune system to the fibrils of the *Limulus* blood clot. Biol Bull 205: 201–203. 14583529 10.2307/1543252

[49] Armstrong PB (2010) Role of α2-macroglobulin in the immune responses of invertebrates. Inv Surv J 7: 165–180.

[50] Lin M, Sutherland DR, Horsfall W, Totty N, Yeo E, et al (2002) Cell surface antigen CD109 is a novel member of the α_2_ macroglobulin/C3, C4, C5 family of thioester-containing proteins. Blood 99: 1683–1691. 11861284 10.1182/blood.v99.5.1683

[51] Cerenius L, Söderhäll K (2011) Coagulation in invertebrates. J Innate Immun 3: 3–8. 21051883 10.1159/000322066

[52] Vargas-Albores F, Hernández-López J, Gollas-Galván T, Montaño-Pérez K, Jiménez-Vega F, et al.. (1998) Activation of shrimp cellular defence functions by microbial products. In: Flegel TW, editors. Advances in shrimp biotechnology. Bangkok: National Center for Genetic Engineering and Biotechnology. pp. 161–166.

[53] Yeh MS, Huang CJ, Cheng JH, Tsai IH (2007) Tissue-specific expression and regulation of the haemolymph clottable protein of tiger shrimp (*Penaeus monodon*). Fish Shellfish Immunol 23: 272–279. 17442588 10.1016/j.fsi.2006.10.007

[54] Armstrong PB, Quigley JP (1985) Proteinase inhibitory activity released from the horseshoe crab blood cell during exocytosis. Biochim Biophys Acta 827: 453–459. 2982412 10.1016/0167-4838(85)90232-8

[55] Armstrong PB (2001) The contribution of proteinase inhibitors to immune defense. Trends Immunol 22: 47–52. 11286692 10.1016/s1471-4906(00)01803-2

